# Predicting Hospitalization for Heat-Related Illness at the Census-Tract Level: Accuracy of a Generic Heat Vulnerability Index in Phoenix, Arizona (USA)

**DOI:** 10.1289/ehp.1307868

**Published:** 2015-01-30

**Authors:** Wen-Ching Chuang, Patricia Gober

**Affiliations:** 1 School of Sustainability, and; 2 School of Geographical Sciences and Urban Planning, Arizona State University, Tempe, Arizona, USA; 3 Johnson-Shoyama Graduate School of Public Policy, University of Saskatchewan, Saskatoon, Saskatchewan, Canada

## Abstract

**Background:**

Vulnerability mapping based on vulnerability indices is a pragmatic approach for highlighting the areas in a city where people are at the greatest risk of harm from heat, but the manner in which vulnerability is conceptualized influences the results.

**Objectives:**

We tested a generic national heat-vulnerability index, based on a 10-variable indicator framework, using data on heat-related hospitalizations in Phoenix, Arizona. We also identified potential local risk factors not included in the generic indicators.

**Methods:**

To evaluate the accuracy of the generic index in a city-specific context, we used factor scores, derived from a factor analysis using census tract–level characteristics, as independent variables, and heat hospitalizations (with census tracts categorized as zero-, moderate-, or high-incidence) as dependent variables in a multinomial logistic regression model. We also compared the geographical differences between a vulnerability map derived from the generic index and one derived from actual heat-related hospitalizations at the census-tract scale.

**Results:**

We found that the national-indicator framework correctly classified just over half (54%) of census tracts in Phoenix. Compared with all census tracts, high-vulnerability tracts that were misclassified by the index as zero-vulnerability tracts had higher average income and higher proportions of residents with a duration of residency < 5 years.

**Conclusion:**

The generic indicators of vulnerability are useful, but they are sensitive to scale, measurement, and context. Decision makers need to consider the characteristics of their cities to determine how closely vulnerability maps based on generic indicators reflect actual risk of harm.

**Citation:**

Chuang WC, Gober P. 2015. Predicting hospitalization for heat-related illness at the census-tract level: accuracy of a generic heat vulnerability index in Phoenix, Arizona (USA). Environ Health Perspect 123:606–612; http://dx.doi.org/10.1289/ehp.1307868

## Introduction

Extreme hot weather events have become life-threatening phenomena in cities around the world (Anderson and Bell 2011; Baccini et al. 2011; Harlan et al. 2013; Loughnan et al. 2013; Sheridan et al. 2012). To estimate the risk of heat-related health consequences and propose adaptation strategies, researchers have developed heat vulnerability indices (HVIs) using composites of health, social, and environmental factors relevant to heat stress (Chow et al. 2012; Johnson et al. 2012; Loughnan et al. 2013; Reid et al. 2009, 2012). Application of HVIs at the neighborhood level allows public-health practitioners and emergency responders to identify and locate populations at high risk of heat stress (Reid et al. 2009). The ability to visualize the spatial variation of heat vulnerability (i.e., on a map) helps local governments allocate resources and assist people in the areas of greatest need. However, human vulnerability to heat is a complex and dynamic issue, and the usefulness of a vulnerability index can be sensitive to scale, measurement, and context. We investigated how generic indicators of heat risk, taken from a national study (Reid et al. 2009) are interrelated in Phoenix, Arizona, and we analyzed the relative importance of different components of Reid et al.’s (2009) national heat-vulnerability index in predicting hospital admissions. Study results may help Phoenix focus its emergency services and climate-adaptation planning on neighborhoods at high risk of heat-related illness and mortality.

## Background

Vulnerability to natural hazards is a function of physical exposure, sensitivity, and adaptive capacity (Chow et al. 2012; Polsky et al. 2007; Turner et al. 2003; Wisner et al. 2004). “Physical exposure” is proximity to environmental hazards, such as heat waves or natural disasters. “Sensitivity” is a characteristic of a population that influences its degree of susceptibility to the hazard, and “adaptive capacity” is the ability to cope with the impacts and aftermath of a hazardous event. Making the concept of vulnerability “operational” has been a challenge because current theoretical concepts and frameworks are abstract and lack guidelines to measure or quantify them (Hinkel 2011). This challenge has stimulated studies to develop measures of vulnerability at various scales (Chow et al. 2012; Harlan et al. 2013; Johnson et al. 2012; Reid et al. 2009, 2012). Research teams with different paradigms have focused on different subsets of vulnerability components (Romero-Lankao et al. 2012), which, in turn, influenced the selection of variables used to evaluate degrees of vulnerability (Tate 2013). Cutter et al. (2003) were perhaps the first to develop a social vulnerability index (SoVI); they used data from the 1990 U.S. Census to examine vulnerability to environmental hazards in 3,141 U.S. counties. This approach to vulnerability indicators (Cutter and Finch 2008) continues to provide the foundation for those seeking indicators of heat risk.

HVI conceptualization and measurement differ from one study to another. In the past decade, at least 13 studies (see Supplemental Material, Table S1) produced different HVIs that revealed the spatial distribution of heat vulnerability for many locations. Most of these studies follow an inductive methodology, which builds statistical models to explain observed harm through some indicating variables (Hinkel 2011; Tate 2013). Researchers select their indicating variables according to empirical analysis (e.g., ethnic minorities are usually more vulnerable than non-Hispanic whites) or social theories (e.g., low social cohesion may negatively affect health) to evaluate an area’s relative risk of heat-related effects. Most studies include as risk factors temperature and vegetation cover (exposure components), age and ethnicity (sensitivity components), and income (adaptive capacity). The concerns of individual disciplines produce different conceptualizations of heat vulnerability indices. For example, environmental modelers (Uejio et al. 2011) have used indicators of the built environment and neighborhood stability to examine heat mortality and heat-related emergency services. They found that neighborhoods with a high proportion of ethnic minorities, social isolation, and vacant housing units had the highest heat-stress incidence. Epidemiologists emphasize health conditions as risk factors—for example, diabetes, which increases susceptibility to heat (Reid et al. 2009, 2012; Rinner 2009). In addition, other variables such as air conditioning (AC) prevalence and social infrastructure (i.e., access to health care facilities) are used as indicators of adaptive capacity in studies of public health, sociology, and epidemiology (Harlan et al. 2013; Loughnan et al. 2013; Reid et al. 2009, 2012). Data from simulation models provide variables to assess future risks to heat. Vescovi et al. (2005) explored the spatial distribution of heat vulnerability in southern Quebec, Canada, under several future climate-change scenarios, using the prediction from the Canadian Regional Climate Model and socioeconomic variables. Each disciplinary perspective captures distinct elements of exposure, sensitivity, and adaptive capacity, and therefore produces varying findings about what determines heat vulnerability.

English et al. (2009) reviewed studies that identified outcomes of climate change and developed indicators for human health vulnerability assessment and found a need to test the usefulness of these indicators. There have been only a few attempts to evaluate the performance of heat vulnerability indices. Wolf and McGregor (2013) used an inductive approach to generate an HVI (and maps) covering 4,765 census units in Greater London, United Kingdom. In their subsequent research, Wolf et al. (2014) validated the performance of their HVI using daily mortality and ambulance dispatch data from 1990 to 2004 and from 1998 to 2006, respectively. The census unit that has an above-average HVI score and an above-average observed health impact score (measured by the number of mortality/ambulance dispatches), and the census unit that has a below-average HVI score and a below-average health impact score are considered as accurate predictions in the work of Wolf et al. (2014). The results showed that the London HVI predicted ambulance calls better than it predicted mortality. London HVI correctly predicted the impacts (measured by ambulance calls) in 3,441 (62.2%) census units during summer days. Wolf et al.’s (2014) findings also suggested that ambulance calls and mortality had different response patterns to heat, consistent with a previous report of contrasting patterns of emergency room admissions and mortality during heat waves in London (Kovats et al. 2004).

In the United States, Reid et al. (2009) developed a national HVI using a statistical approach that integrated factors known to be associated with risk of heat stress in the United States. They selected six sociodemographic and economic indicators (poverty, educational level, minority status, living alone, elderly, and elderly living alone), two AC variables, a measure of vegetation density, and diabetes prevalence to create an HVI for metropolitan statistical areas encompassing 39,794 U.S. Census tracts. They identified four dimensions of heat vulnerability: *a*) social and environmental vulnerability—the aggregation of low education level, poverty, ethnic-minority status, and lack of green space; *b*) social isolation, measured by the proportion of people living alone; *c*) AC prevalence; and *d*) underlying health conditions, represented by the proportion of elderly in the population and the prevalence of diabetes. Later, they asked whether areas with high HVI scores at the ZIP-code scale had higher rates of mortality and morbidity on abnormally hot days (defined by maximum temperature above the 95th percentile for the 30-year temperature distribution) (Reid et al. 2012). They evaluated the relationship in five states: California, New Mexico, Washington, Oregon, and Massachusetts. In California, Washington, and Massachusetts, heat-related illness was more strongly associated with the HVI on abnormally hot days than on other days. But in Oregon, the association between the HVI and heat-related illness did not differ between abnormally hot days and other days. In New Mexico, a 1-unit increase in the HVI was associated with a significant decrease in heat-related hospitalization on abnormally hot days. These findings suggest that local characteristics may influence the accuracy of HVI measures for predicting the risk of adverse heat-related health outcomes in some areas.

Two HVI studies have been conducted in Arizona, using measures similar to those of Reid et al. (2009, 2012). Chow et al. (2012) constructed an HVI using seven indicators from the three dimensions of heat vulnerability (physical exposure, adaptive capacity, and sensitivity) at the census-tract level in metropolitan Phoenix. They used this HVI to investigate geographical change to heat-stress risk between 1990 and 2000, and estimated changes in heat vulnerability among different ethnic populations. They concluded that metropolitan Phoenix had experienced major demographic change during those 10 years, and that demographic change alone had altered the region’s “heatscape.” Harlan et al. (2013) examined neighborhood vulnerability indicators for 2,081 census-block groups in Maricopa County, which includes the Phoenix metropolitan area. Using 278 heat-death cases as dependent variables, they used binary logistic regression to validate a set of HVIs with different combinations of indicators. They concluded that socioeconomic vulnerability, being elderly or isolated, and surface temperature were strong predictors of death from heat exposure.

## Aim and Scope of This Study

Measurement, scale, and context all influence the identification of risk factors. Different combinations of risk factors can produce different “vulnerability landscapes.” To better understand the relationships among risk factors and different scales, we tested Reid et al.’s (2009) national indicators in Phoenix, one of the nation’s hottest cities. We applied Reid et al.’s (2009) variables at the census-tract scale, but measured a few of them differently. We evaluated how accurately the model reflects actual risk of harm locally. At this fine scale, we expected our findings to differ from those of Reid et al. (2009, 2012) and the local research described above (Harlan et al. 2013). We asked where, and what kind of, neighborhoods are at risk of heat-related illness caused by factors beyond social and economic vulnerability, inadequate green space, social isolation, and diabetes. Using a multinomial (polytomous) logistic regression model, with hospital admissions for heat stress modeled as a three-category dependent variable (zero-, moderate-, or high-incidence census tracts), our study explored several questions: *a*) How well does a national HVI explain heat-related hospitalizations in the city of Phoenix? Analyzing the census tracts within the municipal boundary of Phoenix is highly relevant for interventions, because it is the scale at which local governments determine resource allocation and enforce policies. *b*) What is the relative importance of physical exposure, adaptive capacity, and sensitivity to hospitalization incidence, given Phoenix’s hot climate and high prevalence of air conditioning? *c*) In which kinds of neighborhoods is the incidence of heat-related hospitalization explained well or poorly by the HVI? *d*) Are there neighborhood characteristics that are not included in the HVI that predict heat-related hospitalizations in Phoenix?

## Methods

Our dependent variable (hospital admissions for heat stress) came from the Arizona Department of Health Services’ hospital discharge databases for 2004 and 2005. This data set contains a disease code [from the *International Classification of Diseases, 9th Revision–Clinical Modification* (ICD-9-CM)] and the census tract number of the patient’s residence. We used ArcGIS 10 (ESRI, Redlands, CA) to calculate the rate of heat-related illness for each census tract and map 460 heat-related hospitalizations (ICD-9-CM codes 992.0–992.9, effects of heat and light), including heat stroke, heat exhaustion, and other less common heat-related outcomes in 362 census tracts. We normalized the heat-related hospitalizations between 2004 and 2005 by census-tract population estimates for 2010. Rates of hospitalization varied between 0 and 0.76%; the average was 0.03% (see Supplemental Material, Figure S1).

The variables in Reid et al.’s (2009) study were our independent variables. Poverty, low education level, AC prevalence, and social isolation were indicators of adaptive capacity; ethnicity, age, and diabetes prevalence were indicators of a population’s sensitivity to heat; and density of green space indicated both physical exposure and adaptive capacity. Vegetation density has been shown to have a negative relationship with neighborhood temperatures (Jenerette et al. 2007), and it could mitigate the urban heat island effects (Gober 2010; Stone and Norman 2006).

We used data from the 2010 Census (http://factfinder.census.gov/faces/nav/jsf/pages/index.xhtml) for our socioeconomic and demographic variables, which included the percentage of population living below the poverty line (poverty), > 65 years of age (elderly), ethnicity other than non-Hispanic white (minority), having less than a high school diploma (low education), living alone (all ages living alone), and living alone and > 65 years old (elderly living alone) at the census-tract level. To determine poverty, the U.S. Census Bureau uses a set of annual-income thresholds that vary by family size and composition. The poverty threshold for a household in Phoenix with two adults is $14,218 (U.S. Census Bureau 2013a).

We measured diabetes rates differently from Reid et al. (2009, 2012). Whereas Reid et al. (2009, 2012) estimated diabetes prevalence based on age, race, and sex of a county’s population and applied the diabetes incidence rate of each group, we thought this method might miss small-scale effects of diabetes. Thus, we used the diabetes hospitalization rate as an indication of diabetes-related morbidity, and we felt it would provide a better measure of health inequality at the neighborhood level. Using the principal diagnosis code (ICD-9-CM codes 250.0–250.9, diabetes mellitus) and the associated census-tract numbers, we mapped 7,727 cases of diabetes. We used census-tract population estimates for 2010 as the denominator and hospitalizations for diabetes during 2004 and 2005 to calculate census tract–level hospitalization rates for diabetes. The rates varied from 0 to 5.52%; the average was 0.50%. Fifty-three (14.64%) of the census tracts had no hospital admissions for diabetes.

To determine AC prevalence, we aggregated parcel-level residential AC data from the Maricopa County Assessor’s Office to the census-tract level. We obtained vegetation index using a high-resolution (15 m/pixel) ASTER image [NASA Land Processes Distributed Active Archive Center (LP DAAC); https://lpdaac.usgs.gov/data_access]. We combined three images taken on 16 June 2005 and 6 July 2006 to represent Phoenix’s summer vegetation. The Normalized Difference Vegetation Index (NDVI) was calculated using red and near-infrared bands in ERDAS IMAGINE 2011 (download.intergraph.com/downloads/erdas-imagine-2011), a remote-sensing image-processing software.

*Statistical analysis*. A flow chart that illustrates our research steps can be found in Supplemental Material, Figure S2. Factor analysis was conducted using IBM SPSS version 19. We used factor scores from this analysis as independent variables in the multinomial logistic regression (MLR), with health outcomes as dependent variables. A valid regression model that uses geographical/spatial data should consider the effect of spatial autocorrelation/dependency (Ward and Gleditsch 2008). We used global Moran’s *I* to test the distribution of our dependent variable and model residuals. The spatial pattern of the dependent variable was very close to a random distribution (Moran’s *I* = 0.10, *p* = 0.00), and the Moran’s *I* for residuals was 0.02, *p* = 0.00. We divided 362 census tracts into three groups of heat-related health outcomes: zero (146 tracts, 40.33%), moderate (109 tracts, 30.11%), and upper 30th percentile (high incidence, 107 tracts, 29.56%). The deviance and chi-square value are both significant, providing evidence of good fit for the model.

## Results

*Correlation matrix*. Spearman’s correlation coefficients show the relationships among each of the 10 census-tract level vulnerability indicators (Table 1). Diabetes hospitalization rates were significantly and positively correlated with several indicators of socioeconomic disadvantage, including the proportions of the population that were race/ethnicity other than non-Hispanic white, below the poverty line, and that did not have a high school diploma. Reid et al. (2009) found a weaker correlation between diabetes prevalence and these variables (coefficients < 0.3). Use of different methods for the measurement of diabetes and the demographic structure of Phoenix may have affected our findings.

**Table 1:** Spearman’s correlation for vulnerability variables.

Variable	Diabetes	Race/ethnicity other than non-Hispanic white	Age > 65 years	Live alone	Elderly living alone	Below poverty line	Less than high school diploma	Low vegetation	No central AC	No AC of any kind
Diabetes	1.00
Race/ethnicity other than non-Hispanic white	0.63**	1.00
Age > 65 years	–0.13*	–0.54**	1.00
Living alone	0.34**	0.06	0.15**	1.00
Elderly living alone	0.27**	–0.07	0.52**	0.57**	1.00
Below poverty line	0.73**	0.79**	–0.33**	0.31**	0.16**	1.00
Less than high school diploma	0.67**	0.91**	–0.42**	0.09	0.04	0.83**	1.00
Low vegetation cover	0.32**	0.34**	–0.35**	0.12*	–0.03	0.35**	0.38**	1.00
No central AC	0.51**	0.43**	–0.05	0.25**	0.17**	0.51**	0.45**	0.23**	1.00
No AC of any kind	0.52**	0.42**	–0.05	0.26**	0.17**	0.50**	0.43**	0.26**	0.93**	1.00
Spatial unit: census tract; *n* = 362.*p < 0.05. **p < 0.01.										

Another location-specific condition that did not stand out in Reid et al.’s analyses (2009, 2012) is AC prevalence. On the national level, AC variables showed no strong associations (coefficient < 0.02) with poverty and minority status. However, in Phoenix, AC variables have significant positive associations with poverty (coefficient > 0.5) and proportion of minority (coefficient > 0.42). AC is vital to life and comfort in Phoenix, where temperatures average 41°C in July (Cerveny 1996). Although Phoenix’s AC prevalence is > 90%, including central AC and window AC units (U.S. Census Bureau 2013b), the nearly 10% of housing units without AC are concentrated in economically disadvantaged neighborhoods in central Phoenix.

Table 1 also shows that the proportion of elderly was negatively associated with less than high school diploma (–0.42), poverty (–0.33), and low vegetation (–0.35) in Phoenix. These relationships were stronger than the data at the national scale (with coefficients between –0.03 and –0.11). We can therefore interpret that the census tracts in Phoenix with a higher proportion of elderly residents were likely to be wealthier, greener, and better educated than what Reid et al. (2009) found at the county scale for the nation overall. This difference may be attributable to the influx of wealthy retirees into the Phoenix area, and the related proliferation of retirement communities featuring golf courses and outdoor recreational activities (Gober 2006).

**Figure 1:**
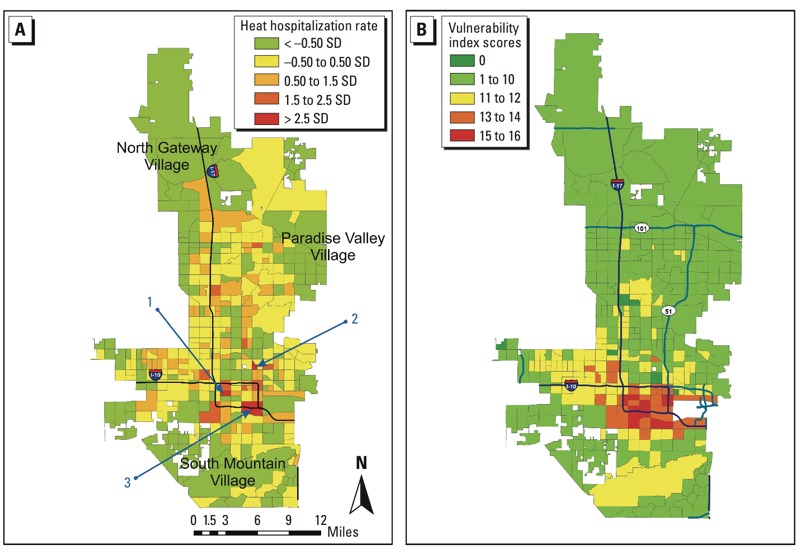
(*A*) Spatial distribution of heat-related hospitalization rate. Census-tract-level hospitalization rates for heat-related illness are hospitalizations for heat-related illness between 2004 and 2005 divided by census-tract population estimates for 2010 times 100 (percent). Nos. 1, 2, and 3 are the top three census tracts with high heat hospitalization rates (> 2.5 SD). (*B*) Heat vulnerability index (HVI; sum of three factor scores) in the city of Phoenix. Each census tract was assigned a score for each factor ranging from 0 to 6 based on SD above or below mean. HVI scores range from 0 to 16.

*Spatial pattern of heat stress in Phoenix*. The map of heat-related hospitalization (Figure 1A) reveals an uneven rate pattern, with higher rates in the urban core. Urban-fringe neighborhoods in northeast, northwest, and south Phoenix had relatively low rates of heat-related hospitalization. Of the three neighborhoods with the highest hospitalization rates, one (no. 3 in Figure 1A), which sits directly west of Sky Harbor Airport, is a low-income neighborhood with a median household income of $20,488 and a Hispanic population of almost 90%. However, the other two (nos. 1 and 2 in Figure 1A) are middle-class (with median household incomes of $40,104 and $37,514) neighborhoods, and Hispanic populations of 25.7% and 52.3%, respectively.

*Factor analysis*. Like Reid et al. (2009), we applied a Varimax rotation in the factor analysis to minimize the number of original variables that load highly on any one factor and increase the variation among factors. We retained three factors (Table 2) with eigenvalues higher than one: *a*) poverty, ethnic minority, and low education; *b*) lack of AC and vegetation; and *c*) diabetes and social isolation, including elderly living alone. Factor 1 explained the highest amount of variance (44.7%); factors 2 and 3 explained 19.98% and 10.46%, respectively. Together they explained 75.14% of the total variance, similar to the results of Reid at al. (2009) in which 75% of total variance was explained by four factors.

**Table 2:** Factor analysis of 10 variables.

Variable	Factor 1	Factor 2	Factor 3
Below poverty line	0.78	0.30	0.32
Race/ethnicity other than non-Hispanic white	0.93	0.10	0.04
Less than high school diploma	0.90	0.18	0.14
Age > 65 years	–0.65	–0.15	0.49
No central AC	0.19	0.92	0.27
No AC of any kind	0.18	0.92	0.27
Low NDVI	0.44	0.45	–0.14
Age > 65 years living alone	–0.06	0.10	0.89
Living alone	0.13	0.23	0.63
Diabetes	0.54	0.27	0.59
Factor 1: poverty, race/ethnic minority and low education; factor 2: lack of AC and vegetation; factor 3: diabetes and social isolation.			

Poverty and minority status are important factors in heat vulnerability, locally and nationally, and are included in factor 1 (see Supplemental Material, Figure S3A). Also included is a negative relationship with elderly populations: Disadvantaged neighborhoods in Phoenix tend to have a large number of children and relatively few elderly residents. Factor 2 combines lack of AC with lack of vegetation, and can be considered a location factor; it is associated with inner-city neighborhoods (see Supplemental Material, Figure S3B). Residents of the inner city are at higher risk from heat than residents elsewhere. Factor 3 (see Supplemental Material, Figure S3C) combines social isolation (especially of elderly people) with diabetes hospitalization. In Phoenix, demographic characteristics make this combination an important factor. The elderly population is at high risk for diabetes (Arizona Department of Health Services 2008). In 2010, according to the U.S. Census Bureau (2011), 121,943 people > 65 years old lived in Phoenix, and about 27% of them lived alone—a higher proportion than in cities neighboring Phoenix. The long history of retirement migration to Phoenix may have resulted in a large proportion of elderly living alone, and this population is at high risk of diabetes hospitalization.

Each census tract was assigned a score for each factor ranging from 0 to 6, where 0 was assigned to tracts with values ≥ 2 SD below the mean for the study area as a whole, and 6 was assigned to tracts with values > 2 SD above the mean. The individual factor scores were then summed to derive the HVI for each tract, with each factor score given an equal weight (Figure 1B), as in other vulnerability studies (Cutter et al. 2003; Harlan et al. 2006; Schmidtlein et al. 2008; Wolf and McGregor 2013). Areas with high HVI scores were clustered in the downtown Phoenix central business district and along the south side of the industrial corridor.

*MLR models*. The results of the MLR show that only factor 1 (poverty and minority status) was a statistically significant predictor (*p* < 0.05) of a moderate-incidence versus zero-incidence tract [odds ratio (OR) = 2.00; 95% confidence interval (CI): 1.50, 2.65 for a 1-unit increase in the factor 1 score)] (Table 3). Factor 1 was also a significant predictor of a high-incidence versus zero-incidence tract (OR = 2.74; 95% CI: 2.03, 3.69), along with factor 3 (OR = 2.00; 95% CI: 1.44, 2.76). These results suggest that census tracts with higher proportions of residents living in poverty and ethnic minorities (factor 1), and tracts with higher rates of hospitalization for diabetes and higher proportions of residents > 65 years of age living alone (factor 3) are more vulnerable to heat stress than other census tracts.

**Table 3:** Odds ratios (ORs) and 95% CIs for associations between a 1-unit increase in each factor and census tracts with moderate or high incidence of hospitalization for heat-related illness relative to zero-incidence census tracts based on multinomial logistic regression.

Predictor	OR (95% CI)	*p*-Value
Moderate-incidence tract
Factor 1	2.00 (1.50, 2.65)	0.00
Factor 2	0.84 (0.56, 1.27)	0.41
Factor 3	1.18 (0.85, 1.64)	0.32
High-incidence tract
Factor 1	2.74 (2.03, 3.69)	0.00
Factor 2	1.20 (0.90, 1.60)	0.21
Factor 3	2.00 (1.44, 2.76)	0.00
Reference category: zero-incidence tract.		

We used the factor scores to predict the category (zero, moderate, and high incidence) of heat-related health outcomes in the MLR model. We then compared the predicted and observed values. From the classification table (Table 4), we found that the scores of HVI did a better job in predicting nonvulnerable areas than vulnerable areas. HVI accurately classified zero-incidence census tracts as zero-incidence tracts 79% of the time, but was less accurate for classifying moderate tracts as moderate versus zero or high incidence (27%) or for classifying high-incidence tracts as high-incidence versus moderate- or zero-incidence (48%). The overall accuracy rate in predicting heat-related outcomes was only 54%, suggesting that accounting for additional factors beyond those in the standard vulnerability index would improve risk prediction.

**Table 4:** Accuracy assessment (classification table).

	Predicted 0	Predicted 1	Predicted 2	Percent correct
Observed 0	115	14	17	78.80
Observed 1	56	29	24	26.60
Observed 2	35	21	51	47.70
Percent correct	56.90	17.70	25.40	53.90
0 = zero-incidence; 1 = moderate-incidence; 2 = high-incidence census tracts.				

Factor 2, with high loadings on lack of AC, was not a significant predictor of heat hospitalization in Phoenix (Table 3). AC has been recommended as a mitigation strategy to reduce heat impacts on health, because many studies find that AC prevalence is negatively associated with adverse health outcomes, especially on extremely hot days (Keatinge 2003; McGeehin and Mirabelli 2001; Semenza et al. 1996, 1999). However, having an AC unit does not automatically mean being able to use it. According to a 2009 survey that interviewed 359 households in three socially vulnerable neighborhoods in Phoenix, many families cannot afford to turn on their AC in the hottest season: 33–50% of respondents who have AC indicated that they avoid using AC to reduce electricity bills (Hayden et al. 2011).

*Unpredictable neighborhoods*. We looked at the neighborhood characteristics of the 14% of census tracts that were oppositely misclassified by the model—35 neighborhoods predicted to be zero-incidence neighborhoods that were actually high-incidence neighborhoods (Table 5, group 1), and 17 neighborhoods predicted to be high-incidence areas that were actually zero-incidence areas (Table 5, group 2). Many group 1 neighborhoods were wealthy neighborhoods on the urban fringe (see Supplemental Material, Figure S4). Group 2 tracts were scattered in central and south Phoenix, and many of them were low-income, and their proportion of Hispanics and the diabetes rate there were higher than the city’s average.

**Table 5:** Characteristics of census tracts with misclassified heat vulnerability based on the HVI compared with average values for all census tracts in Phoenix City.

Characteristic	Group 1 *n* = 35	Group 2 *n* = 17	Phoenix City average *n* = 362
Median household income^*a*^	$52,972	$27,216	$48,750
Non-Hispanic white (%)	97	6	49
Diabetes (%)	34	94	0.5
Noncitizens (%)	11	71	16
Unemployment (%)	23	59	7.5
Proportion of renters (%)	49	65	42
Proportion living in the same residence < 5 years (%)	43	35	46
Vacancy rate (%)	46	71	13
Average surface temperature^*b*^	26.1°C	26.6°C	25.7°C
The percentage for groups 1 and 2 refer to census tracts, not household. The percentages for Phoenix City are citywide average. Group 1: high-incidence census tracts predicted to be zero-incidence census tracts. Group 2: zero-incidence census tracts predicted to be high-incidence census tracts. ^***a***^Group 1: 60% of census tract > average; group 2: 0% of census tract > average. ^***b***^Group 1: 54% of census tract > average; group 2: 76% of census tract > average.			

To better understand risk factors beyond the scope of the national HVI, we looked at variables from other heat vulnerability studies which were not included in the study by Reid et al. (2009). These variables included the size of a census tract’s noncitizen population, the proportion of renters, residents living in the same residence < 5 years, unemployment rate, vacancy rate, and nighttime temperature (Chow et al. 2012; Harlan et al. 2013; Klinenberg 2002). The first variable is a proxy for newcomers who may have limited access to warnings, medical support, and resources that can help them gain relief from heat stress (Chow et al. 2012). Proportions of renters and new residents are measures of population mobility. Short-term renters and newcomers are likely to lack social support and assistance in their neighborhoods (Chow et al. 2012; U.S. Environmental Protection Agency 2006). Unemployment and vacancy rates are typically used as proxies for social stability of a neighborhood; unemployment rate captures the population’s lack of stable economic resources and vacancy rate explains a neighborhood’s prosperity. High unemployment, vacancy, and a high crime rate hinder residents from seeking help in their neighborhoods (Klinenberg 2002). We were not able to acquire crime-rate data at the census-tract scale for Phoenix, but we believe that unemployment and vacancy rates are adequate proxies for social stability. The final factor, nighttime temperature, represents the intensity of the urban heat island effect. We estimated nighttime surface temperatures using the thermal band of three ASTER satellite images (LP DAAC) that cover the entire Phoenix City. The images were taken in June 2003.

The above factors varied widely for the two groups of misclassified neighborhoods, so taking the averages of these variables for the two groups may not adequately represent the groups’ characteristics. We used the city’s average numbers for these variables as thresholds, and calculated the percentage of neighborhoods above the city average for each variable (Table 5). Group 1 neighborhoods, with much higher observed hospital admissions than the HVI predicted, have higher neighborhood mobility (43%) than group 2 neighborhoods (35% mobility). This finding suggests that high neighborhood mobility measured by residency status < 5 years may be associated with higher risk of heat-related illness. In addition, many of the neighborhoods in group 1 were in low-density areas on the urban fringe. The low-density environment offers a different lifestyle than does the urban core, one that may be associated with health outcomes of residents. However, more work is required to understand why these tracts differ from the ones that were better predicted by HVI. Many neighborhoods in group 2 were located in the city core, and had higher population densities and a higher proportion of Hispanic residents than neighborhoods in group 1, but experienced no heat-related hospitalization.

## Discussion

Our findings suggest that low socioeconomic status, as well as the proportion of adults > 65 years of age living alone, percentage of adults living alone, and the rate of hospitalization for diabetes, predict vulnerability to heat at the census-tract level. This finding coincides with studies that found a strong association between poverty, minority, and adverse health outcomes (Curriero et al. 2002; Harlan et al. 2006; Uejio et al. 2011) and studies showing that diabetes was associated with higher risk of heat-related illness (Schwartz 2005; Semenza et al. 1999).

The proportion of dwellings with AC was not a significant predictor of heat-related hospital admissions in Phoenix, perhaps because the incidence of AC is so high or because having AC does not imply using it. Some heat-related illness occurs in those who work outside or engage in outdoor activity. Thus, having an AC at home does not eliminate the risk of heat-related health problems. Therefore, reducing the risk of heat-related hospitalizations requires more than increasing home AC units. It also requires *a*) more effective risk mitigation for people who work or recreate outside; *b*) identification of socially isolated, diabetic patients; and *c*) awareness of the concentration of effects in disadvantaged neighborhoods.

From a political economic perspective, the process of marginalization is a fundamental factor making some urban residents (i.e., low income) more vulnerable to natural or environmental hazards (Browning et al. 2006; Klinenberg 1999). However, there are other social characteristics, such as social capital or social networks, not measured by common social vulnerability indicators, that could offset the impact of environmental hazards on low-income or minority populations (Romero-Lankao et al. 2012). Several studies have found that some socioeconomically disadvantaged groups and immigrants have strong internal social networks that foster social cohesion and fast recovery from disasters (Chamlee-Wright and Storr 2009; Klinenberg 2002; Li et al. 2010). Klinenberg (2002) suggested that strong social networks, pedestrian-friendly streets, and shops, restaurants, and community organizations are sources of resilience that can save lives from heat stress. Living in a neighborhood with a robust social infrastructure that provides an environment for mutual assistance could reduce negative health impacts, especially during disasters (Sampson 2011).

High socioeconomic status does not necessarily mean low heat vulnerability, and vice versa. Our misclassified neighborhoods included wealthy, non-Hispanic white neighborhoods with higher hospital admissions than the HVI would have predicted. Many of these neighborhoods had a higher proportion of households that have relocated to the neighborhood in the past 5 years than the city average. Programs that enhance residents’ awareness of heat risks might also reduce the incidence of negative health outcomes in transient neighborhoods.

Our findings provide information that can help the city government plan effective interventions. We recommend a two-stage strategy to reduce heat-related hospital admissions in Phoenix. The first stage should focus on immediate and short-term heat-mitigation among socioeconomically disadvantaged populations, especially in central Phoenix. We suggest that the municipal government relocate resources to neighborhoods with high HVI scores in the urban core. Interventions might include opening cooling centers during extreme heat events, providing information about how to prevent heat-related illness to disadvantaged populations, and increasing the efficiency and affordability of residential AC. The second-stage policy should focus on long-term planning. Because high social isolation is associated with higher risk of heat-related illness, programs to care for people living alone or making warning information accessible to those living alone are likely to reduce heat-related hospital admissions.

Good planning practices that improve health can bring co-benefits to the residents. It has been shown that changing urban design to reduce automobile dependency and carbon dioxide emissions (for example, creating a comfortable, pedestrian-friendly environment that increases walkability in neighborhoods) can also reduce the risks of cardiovascular disease, obesity, and diabetes (Lathey et al. 2009), all of which that exacerbate the outcomes of heat stress.

We acknowledge that this study has several limitations. For the present analysis we used ICD-9-CM code 992 as the only outcome because this category is a straightforward measurement of heat impact on human health. However, using only this data set, we might underestimate heat impacts on human health because *a*) this data set records only serious cases that require hospitalization, and, *b*) there are other human-health problems relevant to excessive heat, such as cardiovascular disease and respiratory diseases (Reid et al. 2012). The second limiting factor is that we assume the heat-related illness will have an equal probability of resulting in hospitalization in any census tract. However, compared with other residents, low-income people without health insurance and non-U.S. citizens may be less likely to seek medical care, and less likely to be hospitalized if they do seek care, even if they have the same severity of heat-related illness. Furthermore, the neighborhood mobility indicators (e.g., residence for < 5 years) may not necessarily represent the actual social conditions, such as lack of social cohesion. Moreover, data used to characterize the predictors and the outcomes are defined at the census-tract scale. Although group-level associations are informative and relevant for planning group-level interventions, associations with group-level characteristics cannot be assumed to represent associations with the same characteristics defined at individual level. Last, we used 2010 U.S. Census data to define HVI, but health outcomes were from 2004 to 2005. Although this may not result in substantial bias or misclassification, this remains a potential limitation.

## Conclusions

Generic indicator systems can predict the risk of heat-related health problems adequately and provide a useful picture of the spatial distribution of risk, but they are sensitive to scale, measurement, and context. Decision makers need to reflect on the particular characteristics of their cities to determine how well the vulnerability maps reflect actual risk of harm. In Phoenix, the variables used on a national scale allowed us to accurately classify only about 54% of the census tracts based on heat hospitalizations. There is, however, a larger story about heat stress that is not captured by the standard vulnerability measures. There is no one-size-fits-all vulnerability indicator. Different types of problems and concerns require multiple strategies to evaluate the degree of vulnerability. Our study demonstrated that researchers need to take into account the wide institutional and social context that determines vulnerability, as expressed by the concept of “contextual vulnerability” (Hinkel 2011). In addition, vulnerability studies should not be limited to just the identification of vulnerable people and places, but should also include the exploration of the sources of resilience in communities. Further research can build upon our heat vulnerability map to identify the source of resilience to heat in Phoenix and further investigate the factors that put neighborhoods at risk of heat-related illness.

## Supplemental Material

(560 KB) PDF
