# Supplementary material for: Predicting Hospitalization for Heat-Related Illness at the Census-Tract Level: Accuracy of a Generic Heat Vulnerability Index in Phoenix, Arizona (USA)
Source: Environ Health Perspect. 2015 Jan 30;123(6):606–12. doi: 10.1289/ehp.1307868 (PMC4455581; doi:10.1289/ehp.1307868)
Supplement: (560 KB) PDF [file ehp.1307868.s001.508.pdf]

Environmental Health Perspectives doi:10.1289/ehp.1307868

Supplemental Material

Predicting Hospitalization for Heat-Related Illness at the Census Tract Level: Accuracy of a Generic Heat Vulnerability Index in Phoenix, Arizona (USA)

Wen-Ching Chuang and Patricia Gober

Table S1. Literature that conceptualized and visualized vulnerability with geospatial techniques.

Reference	Approach/method	Variables (measures)	Study area	Spatial unit	Evaluate HVI with health data?
Vescovi et al. 2005	Integrated climate variables and socio-economic parameters in GIS to produce maps that estimate present and future public health risk to excessive heat.	Temperatures, proportion of residents > 65 years old, poverty, social isolation (proportion of single person household), education level.	Southern Quebec, Canada	Census subdivision	No
*Lindley et al. 2006	Proposed a framework/method to visualize areas that are vulnerable to heat hazards, and project risks to heat.	Maximum temperature, population > 75 years of age living alone, population < four years old, population with chronic illness, population with mental health problems or is bedridden, income disparity, land-use type.	Greater London, the United Kingdom	Census block	No
*Reid et al. 2009	Used factor analysis to analyzed 10 heat vulnerability indicators, and calculated HVI using the sum of factor scores.	Prevalence of diabetes, race other than white, population > 65 years old, living alone, population > 65 years old and living alone, population below poverty line, population without high school diploma, no green space, no central AC, no AC any kind.	Metropolitan statistical areas, USA	Census tract	No
*Rinner et al. 2009	Proposed 14 measures that represent exposure, sensitivity, and adaptive capacity to assess potential vulnerability to heat.	Remotely sensed land surface temperature, lack of tree canopy, green space, old dwellings without AC, high-density dwellings without AC, behavior, pre-existing/chronic illness, cognitive impairment, elderly residents, infants and young children, low-income households, rental households, socially isolated people, homeless, low education level, population not speaking English, recent immigrants, racialized groups, access to cooling centers.	Toronto, Canada	Census tract	No
Chow et al. 2012	Constructed a HVI by combining the normalized scores of five socioeconomic variables and three environmental indicators.	Summer temperatures, vegetation index, proportion of residents > 65 years old, median household income, proportion of foreign-born noncitizens, proportion living in the same house < five years.	Metropolitan Phoenix area, AZ, USA	Census track	No
Uejio et al. 2011	Used Generalized Linear and Mixed Models to identify risk factors of heat vulnerability linked to heat mortality or morbidity.	Selected 22 variables, including vegetation index, remotely sensed surface temperatures, impervious surface, housing density, single family detached homes, poverty, households renting, proportion of residents > 65 years old, proportion living alone, proportion of people with disabilities, linguistically isolated households, household with more than seven residents, percent ethnic minorities, proportion living in the same house < five years, vacant households, house age, housing value. Their statistical results suggested four and 13 significant factors for Philadelphia and Phoenix respectively.	Philadelphia, PA; metropolitan Phoenix, AZ, USA	Census block groups	Heat-mortality identified by The Philadelphia Department of Health between July 8 and August 4, 1999 (n=64). Heat-related emergency calls identified by the City of Phoenix Regional Fire Department Dispatch Center between June and September, 2005 (n=637).
*Reid et al. 2012	Used Poisson regression to relate HVI to heat and non-heat-related health conditions during extremely hot days in five states in the USA.	Prevalence of diabetes, race other than white, proportion of residents > 65 years old, living alone, population > 65 years old and living alone, population below poverty line, population without high school diploma, no green space, no central AC, no AC any kind.	California, New Mexico, Washington, Oregon and Massachusetts, USA.	Zip-code area	Counts of hospital admission for electrolyte imbalance, cardiovascular, cerebrovascular disease, respiratory illness, nephritis and nephrotic syndrome, acute renal failure, heat-related illness, and internal causes of hospitalization, and number of daily mortality.

Reference	Approach/method	Variables (measures)	Study area	Spatial unit	Evaluate HVI with health data?
Johnson et al. 2012	1) Used factor analysis to build heat vulnerability index (EHVI) from 15 census 1990 variables, and three environmental indicators; 2) used the add-up EHVI factor scores to test the effectiveness of the EHVI in explaining death rates during excessive heat events.	28 variables were presented. Only 19 variables were used for the construction of EHVI: females > 65 years old, males > 65 years old, females > 65 years of age and living alone, males > 65 and living alone, white population, females head of household, mean family income, per capita income, mean household income, population > 25 years old and without high school diploma, Asian population, proportion of residents > 65 years old and living alone, other race population, Hispanic population, population > 25 years old with a high school education, built-up index, vegetation index, Black population, remotely sensed land surface temperature.	Chicago, IL, USA	Census block group	Heat mortality data from death certificate during a heat wave in July 1995 (n=586). Residential heat death is defined by the Illinois State Vital Records Department.
Harlan et al. 2013	1) Used factor analysis to construct a set of HVIs from U.S. Census data and remotely sensed vegetation and land surface temperature; 2) used binary logistic regression and spatial analysis to associate heat-related death with HVI.	ethnic minority, Latino immigrant, population below poverty line, population without high school diploma, proportion of residents > 65 years old, proportion of residents > 65 years of age living alone, population living alone, no air conditioning, unvegetated area, remotely sensed surface temperature.	Maricopa County, AZ, USA	Census block group	Heat-mortality data from death certificate (n=278) from 2000 to 2008. Heat death is identified by a surveillance system specifically designed to identify heat-caused and heat-related deaths associated with weather in Maricopa County.
Hondula et al. 2012	1) Used randomization test to identify mortality exceedances for several apparent temperature thresholds; 2) used factor analysis to identify the environmental, demographic and social factors associated with high-risk areas.	25 explanatory variables from five aspects: zoning and land use, social demographics, income level, and remotely sensed land surface temperature.	Philadelphia County, PA, USA	Zip code area	All-cause mortality records from 1983 to 2008 (n=409,554).
Loughnan et al. 2013	1) Identified threshold temperatures at which risk of mortality or morbidity increase in eight Australian cities; 2) used factor analysis to derived vulnerability index; 3) used climate model output to predict changes of days with excessive heat; 4) estimated changes in risk related to changing population density and aging population.	populations < 4 and > 65 years old, aged care facilities, socioeconomic status, urban design (non-single dwellings), proportion of single-person households, population need for assistance (disability), population density, ethnicity, remotely sensed surface temperature, land cover, accessibility to emergency service.	Brisbane; Canberra; Darwin; Hobart; Melbourne; Perth; Adelaide; Sydney, Australia	postal area	No
*Wolf et al. 2013<br><br>*Wolf et al. 2014	1) Derived a HVI using factor analysis of nine proxy measures of heat risk; 2) discussed drivers of uneven spatial patterns of heat vulnerability.<br><br>Examine the performance of the HVI on heat-wave days and non-heat-wave days.	Households in rented tenure, household in a flat (one-storey), population density, household without central heating, population > 65 years old, aged population with long-term limiting illness, population with self-reported health status "not good", receive any kind of social benefit, single pensioner household.	London, United Kingdom	Census district	No<br><br>Mortality and ambulance callout data from 1990 to 2006.

*Including at least one health variable (preexisting health condition) as a component of HVI.

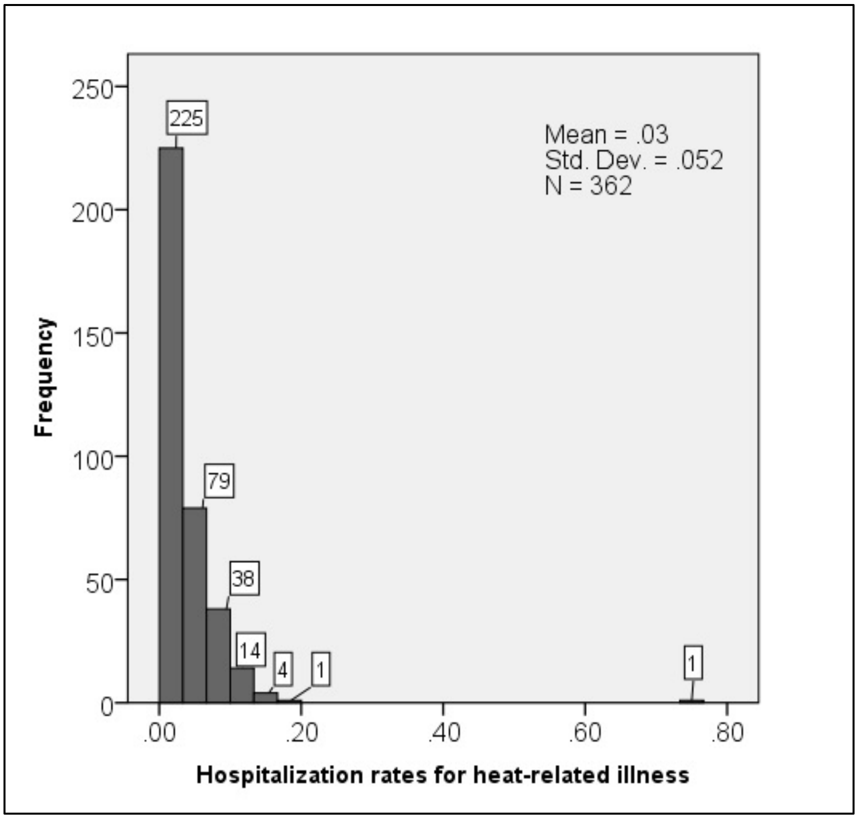

Figure S1. The distribution of the hospitalization rates for heat-related illness, positively skewed, with a mode of 0, median of 0.02, mean of 0.03, and standard deviation of 0.05.

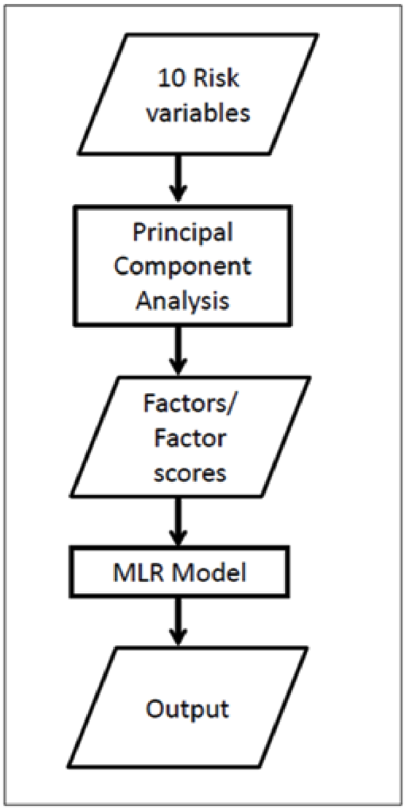

Figure S2. A flow chart of the research procedures.

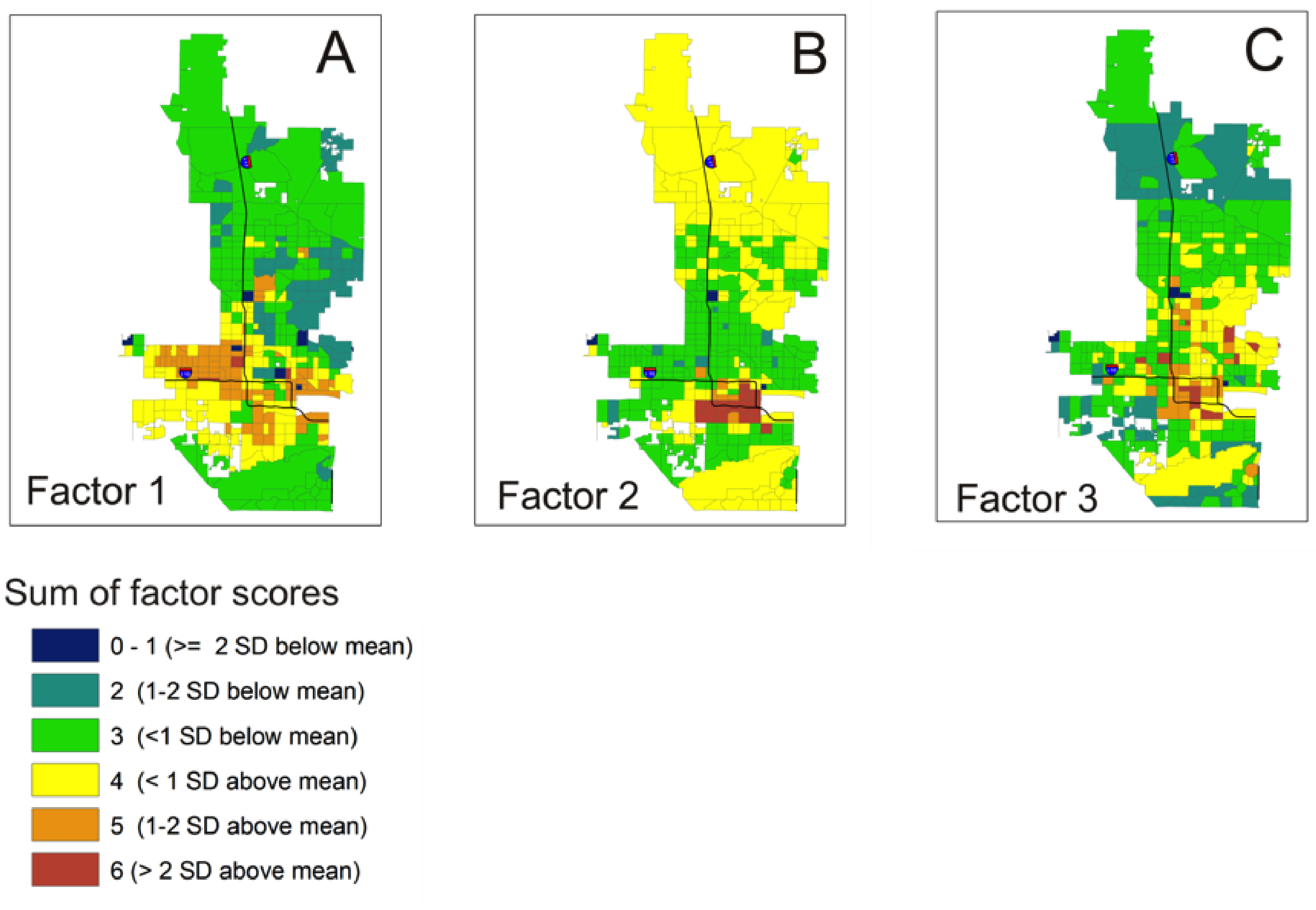

Figure S3. Maps of individual factor scores. (A) Factor 1: Poverty, ethnic minority, and low-education level; (B) Factor 2: lack of AC and vegetation; (C) Factor 3: diabetes and social isolation.

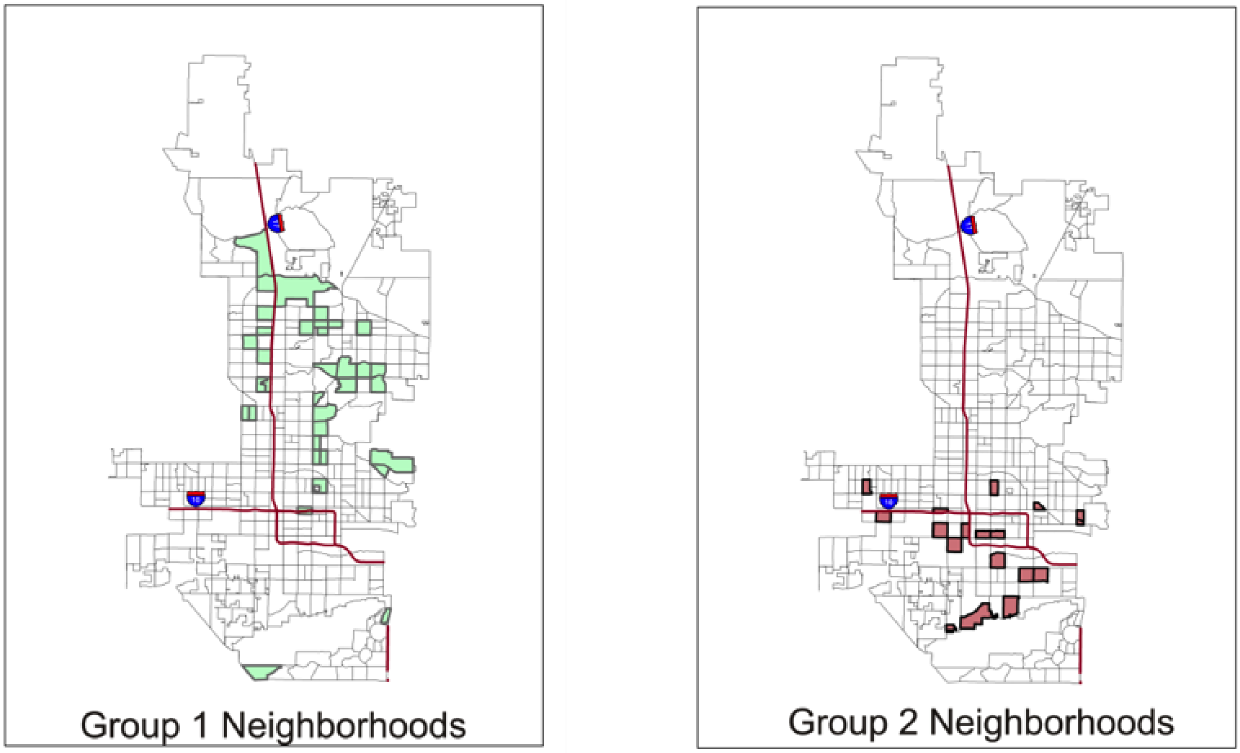

Figure S4. Neighborhoods that are misclassified in the MLR model: (A) The high-incidence neighborhoods that are predicted as zero-incidence neighborhoods. (B): The zero-incidence neighborhoods that are predicted as high-incidence neighborhoods.

References

Chow WTL, Chuang W, Gober P. 2012. Vulnerability to extreme heat in metropolitan Phoenix: Spatial, temporal, and demographic dimensions. The Professional Geographer 64(2):286-302; doi: 10.1080/00330124.2011.600225.

Harlan SL, Declet-Barreto JH, Stefanov WL, Petitti DB. 2013. Neighborhood effects on heat deaths: Social and environmental predictors of vulnerability in Maricopa County, Arizona. Environ Health Perspect 121(2):197-204; doi: 10.1289/ehp.1104625.

Hondula D, Davis R, Leisten M, Saha M, Veazey L, Wegner C. 2012. Fine-scale spatial variability of heat-related mortality in Philadelphia county, USA, from 1983-2008: A case-series analysis. Environ Health 11(1):16.

Johnson DP, Stanforth A, Lulla V, Luber G. 2012. Developing an applied extreme heat vulnerability index utilizing socioeconomic and environmental data. Appl Geogr 35(1–2):23-31; doi: 10.1016/j.apgeog.2012.04.006.

Lindley SJ, Handley JF, Theuray N, Peet E, Mcevoy D. 2006. Adaptation strategies for climate change in the urban environment: Assessing climate change related risk in UK urban areas. Journal of Risk Research 9(5):543-568.

Loughnan M, Tapper N, Phan T, Lynch K, McInnes J. 2013. A spatial vulnerability analysis of urban populations during extreme heat events in Australian capital cities. Gold Coast: National Climate Change Adaptation Research Facility.

Reid CE, O'Neill MS, Gronlund CJ, Shannon J. Brines, Brown DG, Diez-Roux AV et al. 2009. Mapping community determinants of heat vulnerability. Environ Health Perspect 117(11):1730-1736.

Reid C, Mann J, Alfasso R, English P, King G, Lincoln R et al. 2012. Evaluation of a heat vulnerability index on abnormally hot days: An environmental public health tracking study. Environ Health Perspect 120(5):715-720; doi: doi: 10.1289/ehp.1103766.

Rinner C. 2009. Development of a Toronto-Specific, Spatially Explicit Heat Vulnerability Assessment [Electronic Resource] Phase I Final Report. Toronto (Ont.) Dept of Public Health.

Uejio CK, Wilhelmi OV, Golden JS, Mills DM, Gulino SP, Samenow JP. 2011. Intra-urban societal vulnerability to extreme heat: The role of heat exposure and the built environment, socioeconomics, and neighborhood stability. Health Place 17(2):498-507.

Vescovi L, Rebetez M, Rong F. 2005. Assessing public health risk due to extremely high temperature events: Climate and social parameters. Climate Research 30(1):71; doi: 10.3354/cr030071.

Wolf T, McGregor G. 2013. The development of a heat wave vulnerability index for London, United Kingdom. Weather and Climate Extremes 1(0):59-68; doi: http://dx.doi.org/10.1016/j.wace.2013.07.004.

Wolf T, McGregor G, Analitis A. 2014. Performance assessment of a heat wave vulnerability index for greater London, United Kingdom. Wea Climate Soc 6(1):32-46; doi: 10.1175/WCAS-D-13-00014.1.
